# The Effects of Fatiguing Aerobic Exercise on the Cerebral Blood Flow and Oxygen Extraction in the Brain: A Piloting Neuroimaging Study

**DOI:** 10.3389/fneur.2019.00654

**Published:** 2019-06-21

**Authors:** Dapeng Bao, Junhong Zhou, Ying Hao, Xuedong Yang, Wei Jiao, Yang Hu, Xiaoying Wang

**Affiliations:** ^1^Sport Science Research Center, Beijing Sport University, Beijing, China; ^2^The Hinda and Arthur Marcus Institute for Aging Research, Hebrew SeniorLife, Roslindale, MA, United States; ^3^Harvard Medical School, Boston, MA, United States; ^4^Peking University, Academy for Advanced Interdisciplinary Studies, Beijing, China; ^5^Department of Radiology, Peking University First Hospital, Beijing, China

**Keywords:** aerobic exercise, fatigue, fMRI, cerebral blood flow, oxygen extraction fractions

## Abstract

The fatigue in aerobic exercise affects the task performance. In addition to the fatigue in the muscular system, the diminished performance may arise from the altered cerebral blood supply and oxygen extraction. However, the effects of the fatiguing aerobic exercise on the ability of brain to regulate the cerebral blood flow (CBF) and to extract the oxygen are not fully understood. In this pilot study, we aim to quantify such effects via advanced functional MRI techniques. Twenty healthy younger elite athletes were recruited. In the screening visit, one circle ergometer test was used to screen the maximal relative oxygen consumption (V_O2max_). Eleven eligible participants then completed the next MRI visit after 7 days. These participants completed a 2-min pulsed arterial spin labeling (ASL) using the PICORE/QUIPSS II and 5-min asymmetric spin echo (ASE) scan at baseline and immediately after the aerobic circle ergometer test. The CBF was then measured using the ASL images and the oxygen consumption of the brain was quantified using oxygen extraction fractions (OEF) derived from the ASE images. The test time, V_O2max_, and anaerobic threshold were also recorded. As compared to baseline, participants had significant reduction of global CBF (*p* = 0.003). Specifically, the CBF in bilateral striatum, left middle temporal gyrus (MTG) and right inferior frontal gyrus (IFG) decreased significantly (*p* < 0.005, *K* > 20). No significant changes of the OEFs were observed. Participants with greater OEF within the right striatum at baseline had longer test time, greater anaerobic threshold and relative V_O2max_ (*r*^2^ > 0.51, *p* < 0.007). Those with longer test time had less reduction of CBF within the right IFG (*r*^2^ = 0.55, *p* = 0.006) and of OEF within the left striatum (*r*^2^ = 0.52, *p* = 0.008). Additionally, greater anaerobic threshold was associated with less reduction of OEF within the left MTG (*r*^2^ = 0.49, *p* = 0.009). This pilot study provided first-of-its-kind evidence suggesting that the fatiguing aerobic exercise alters the cerebral blood supply in the brain, but has no significant effects on the ability of brain to extract oxygenation. Future studies are warranted to further establish the CBF and OEF as novel markers for physical and physiological function to help the assessment in the sports science and clinics.

## Introduction

Physiological fatigue is one of the main contributors to the diminished performance in aerobic exercise. With the increase of the exercise load, multiple physiological factors (1, 2), such as the intramuscular metabolism, excitation-contraction coupling, are altered, causing the inability of muscles to produce enough power forming the voluntary motion (3). In addition to these peripheral factors, the fatigue may affect the functionality of the brain, including the regulation of cerebral hemodynamics and oxygen consumption (4).

The successful completion of the motion in exercise is dependent upon the capacity of neurons in the brain to process the afferent information from peripheral systems and send the feedback to musculoskeletal system via neurotransmitters appropriately. These important neural activities rely on the sustainable supply of the oxygenated blood and the extraction of oxygen. Previous studies have provided preliminary evidence showing the effects of the exercise-induced fatigue on the cerebral hemodynamics and oxygen consumption. Poulin et al. (5), for example, observed the global cerebral blood flow (CBF) of the brain, as measured using transcranial Doppler ultrasound (TCD), increased after the exercise of mild load (i.e., at 20 and 40% of the maximal oxygen uptake). In a separate study, Thomas and Stephane (6) observed that the oxygenation of the prefrontal cortex, as measured using near-infrared spectroscopy (NIRs), increased in the first minutes of the exercise but decreased when the exercise load increased exhaustively. However, the changes of the CBF and the extraction of the oxygen within the specific brain regions, as well as the underlying mechanism of such regulation in response to aerobic exercise (5), are still unclear.

The advanced functional magnetic resonance imaging (fMRI) techniques enable non-invasively quantifying the functional characteristics of the brain, including the cerebral blood flow and oxygen consumption with high-resolution images. The development of the oxygen extraction fraction (OEF) sequences in fMRI (7), for example, allows measuring oxygen uptake of the small brain regions (as small as within several voxels). In this pilot study, we aim to explore the effects of fatiguing aerobic exercise on the regulation of cerebral blood flow and the oxygen extraction of the brain via these advanced fMRI techniques. Specifically, we hypothesize that after the aerobic circle ergometer exercise with incremental load to the maximal oxygen consumption (V_O2max_), participants would have a significant decrease in their CBFs and OEFs, particularly in the cerebral regions associated with the voluntary movements and task motivation.

## Methods

### Participants

After screening the performance of 800-meter race in 410 healthy young athletes, 20 of them were recruited in this study. All of them were “elite” as they were able to complete the 800-meter race within 123 s. They had no injury within the past 3 months and were without any self-reported and/or diagnosed metabolic or neurological diseases. Those with a relative V_O2max_ <55 ml/min/kg (8–10), as measured in the screening visit, were excluded for the following MRI test.

## Ethics Statement

This study was approved by Institutional Review Board of Beijing Sport University, and conducted according to the principles of the Declaration of Helsinki. All the participants provided written informed consent as approved by the institutional review board.

### Study Protocol

This study consisted of two visits: the screening visit and MRI visit. The participants were first screened based upon their relative V_O2max_ measured in the cycle ergometer test in the screening visit. Eligible participants then completed the MRI visit 7 days after and the CBF and OEF were measured during the MRI visit.

### Screening Visit

All the twenty participants completed one cycle ergometer test on an electrical cycle ergometer (Monark839) on this visit. They were instructed to not perform heavy exercise at least 24 h prior to the visit or any other exercise on the same day of the visit. They were also asked to not take consuming food or beverages containing caffeine before the test. The temperature in the testing room was maintained between 20 and 25°C, and the relative humidity was between 40% and 50%. The load of the ergometer was set at 90 Watts at the beginning of the test and increased progressively with 15 Watts per minute. The gas analyzer (AEI Technologies, TX USA) was used to assess the oxygen consumption. Before each test started, the gas analyzer was calibrated. Several metrics of the oxygen consumption, including the relative V_O2max_ and anaerobic threshold, were measured. The study personnel used the Borg Rating of Perceived Exertion scale to assess the degree of the fatigue of the participants during the test (11). This scale scored from 6 to 20 and greater score represented higher fatigue. When the participant reported a score ≥19 (i.e., the exercise load with their maximal effort), the test stopped. Nine of the participants had the relative V_O2max_ <55 ml/min/kg and were thus excluded.

### MRI Visit

The 11 eligible participants then completed the MRI visit 7 days after the screening visit. All the participants completed the MRI scan, consisting of a 2-min pulsed arterial spin labeling (ASL) scan (12, 13) and a 5-min asymmetric spin echo (ASE) scan (14) before and immediately after the cycle ergometer test. The same protocol of ergometer test as in the screening visit was used. The duration of the cycle ergometer test (i.e., test time), relative V_O2max_ and anaerobic threshold was recorded and used in the following analyses.

A 3T MRI scanner (GE Medical System) with 8-channel standard head array coil was used to acquire the MRI data. A 3D FSPGR scan was collected for whole-brain high-resolution anatomy. The ASL was used to acquire the CBF using the following parameters: PICORE/QUIPSS II; Slice thickness/gap = 8.0/2.0 mm; flip angle = 90 degrees, field of view = 230 mm × 230 mm, TR = 3,000 ms; TE = 3.1 ms, TI_1_ (inversion time) = 700 ms, TI_2_ = 1,500 ms; volumes = 50. The ASE protocol, consisting of one spin echo and 19 ASE scans, was used to acquire the OEF (TE = 65 ms, TR = 3,000 ms, field of view = 240 mm × 240 mm, 64 × 64 acquisition matrix, slice thickness = 5 mm, 20 slices, Nex = 2, Tau = 49).

### Data Processing

The CBF data were pre-processed using Statistical Parametric Mapping software (SPM8, Wellcome Department of Imaging Neuroscience, University College, London, UK) and ASLtbx (15, 16). The motion artifact was removed first using the realignment function. Specifically, the rigid body transform was used to estimate the motion time courses for all ASL's control and label images. Sinc interpolation of the ASL was then used to avoid the BOLD contamination. The time-matched control and label images were created, followed by subtraction to suppress BOLD contamination (16, 17). The CBF image series were generated based on a single compartment continuous ASL perfusion model using ASLtbx (16). Functional images were reoriented with the origin (i.e., the coordinate of *x* = 0, *y* = 0, and *z* = 0) set at the anterior commissure. Then the ASL images were co-registered to the corresponding anatomical images and then normalized to the MNI (Montreal Neurological Institute) space for group analysis (16). The registration performance of images was visually checked. These data were then smoothed using a Gaussian kernel of full-width half-maximum 8 mm. The CBF maps were constructed using an in-house program by applying a gray matter mask for the calculation, and the threshold (i.e., probability of gray matter) was set>0.8. The global and the regional CBFs of cluster with a size of at least 20 voxels were then obtained following the method proposed by Wang et al. (16).

The ASE data were acquired using different times ranging from 10 to 24 ms with an increment of 0.5 ms. These data were processed using a customized Matlab (MathWorks Inc. Natick, MA, USA) program (14, 18). To improve the signal-to-noise ratio, all the ASE images were first filtered using Gaussian low-pass filter (the kernel size was 3 × 3, and the standard deviation was 1.5).

The global and regional OEFs were then obtained using the method proposed by An and Lin (7). Specifically, the measurement of OEF and R2′ was derived from a theoretical model proposed by Yablonskiy and Haacke (19), in which a set of randomly orientated cylinders was used to characterize the signal behavior in static dephasing regime. The signal can be written as:

$$\begin{array}{l} S\left(\tau \right)=\rho \left(1-\lambda \right)\cdot \text{f}\left(\lambda ,\delta \omega ,\tau \right)\cdot \left(-\frac{TE}{T2}\right)\cdot g\left(\tau ,T1,TR\right), \end{array}$$

where ρ was the effective spin density, λ was the volume fraction of muscle occupied by deoxyhemoglobin; where δω was the characteristic frequency shift and was defined as:

$$\begin{array}{l} S\omega =\frac{4}{3}\pi \cdot \gamma \cdot \;\Delta \chi_{0}\cdot H_{ct}\cdot B_{0}\cdot OEF, \end{array}$$

where γ was the gyromagnetic ratio; Δχ_0_was the susceptibility difference between the fully oxygenated and fully deoxygenated blood; H_ct_ was the fractional hematocrit, B_0_ was the main magnetic field strength, and Δχ_0_ of 0.27 ppm per unit H_ct_ in centimeter-gram-second units.

### Statistical Analysis

All the statistical analyses were performed by using the Matlab. To examine the effects of fatiguing aerobic exercise on the CBF, we first identified the regions with significant changes in CBF after the circle ergometer test using the paired-*t* test to compare the CBFs within each voxel before (i.e., baseline) and after the test. The significance level here was set as *p* < 0.01, and the threshold of cluster size (i.e., K) was set as 20. Then we further corrected the multi-comparison results using the false discovery rate (FDR). To examine the effects of fatiguing aerobic exercise on the OEF, we compared the global OEFs and regional OEFs before and after the test. Particularly, we focused on those FDR-identified regions with significant change in CBFs [i.e., regions of interest (ROIs)]. The FDR was also used to compare the OEF within the ROIs before and after the test. Secondarily, the association between the oxygen consumption (i.e., the V_O2max_, anaerobic threshold), the test time and the regional CBFs and OEFs were examined using linear regression. The Bonferroni correction was used in the multiple comparison.

## Results

All the 11 participants [age: 20.3 ± 0.8 (mean ± S.D.) years, BMI: 21.6 ± 1.6] completed the circle ergometer test and two MRI scans. The time of the cycle ergometer test they maintained was 687 ± 72.1 s. The relative V_O2max_ was 60.1 ± 3.3 ml/min/kg and the anaerobic threshold was 2882.3 ± 245.4 ml/min as measured by the gas analyzer.

**Figure 1:**
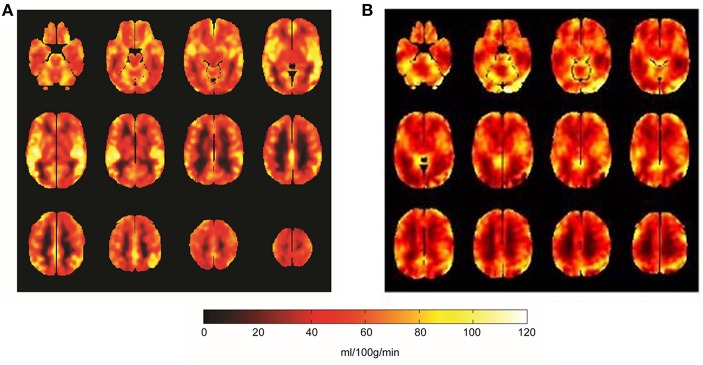
The global CBF mappings at baseline **(A)** and after the fatiguing aerobic exercise **(B)**. Compared to baseline, the global CBF across the brain significantly decreased (*p* = 0.003) after the aerobic exercise. Brighter color in the figure was greater CBF (unit: ml/100 g/min).

**Figure 2:**
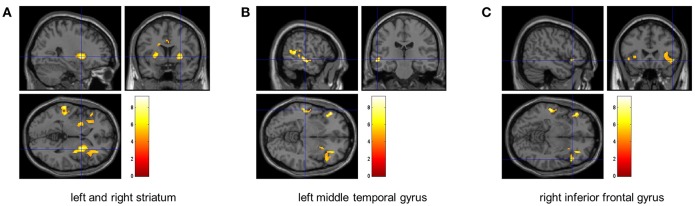
The identified brain regions with significant decrease of CBF. The FDR analyses revealed that the CBF within left and right striatum (left: *p* = 0.004, right: *p* = 0.005) **(A)**, left middle temporal gyrus (*p* < 0.0001) **(B)**, and right inferior frontal gyrus (*p* = 0.005) **(C)** significantly decreased after the fatiguing aerobic exercise as compared to baseline.

Compared to the baseline, significant reduction in the global CBF was observed after exercise (*p* = 0.003, Figure 1). The CBFs in four brain regions, including left and right striatum, left middle temporal gyrus (MTG) and right inferior frontal gyrus (IFG), significantly decreased after completing the cycle ergometer test (*K* > 20, *p* < 0.005, Figure 2). However, no significant changes were observed in global and regional OEFs as compared to the baseline (*p* > 0.21, Table 1).

**Table 1:** The regional OEFs before and after the cycle ergometer test.

**Regions**	**Baseline**	**After the test**	***p-*values**
Global	0.34 ± 0.1	0.33 ± 0.09	0.52
L-striatum	0.16 ± 0.10	0.13 ± 0.08	0.21
R-striatum	0.23 ± 0.04	0.22 ± 0.07	0.56
L-middle temporal gyrus	0.12 ± 0.07	0.13 ± 0.09	0.65
R-inferior frontal gyrus	0.10 ± 0.05	0.11 ± 0.07	0.66

**Figure 3:**
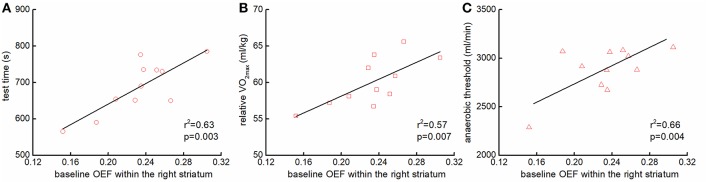
The association between the baseline regional OEFs and functional performance. Participants with greater OEF within the right striatum at baseline sustained the cycle ergometer test longer [*r*^2^ = 0.63, *p* = 0.003; **(A)**], and had greater the relative maximal oxygen consumption (VO_2max_) [*r*^2^ = 0.51, *p* = 0.007; **(B)**], and anaerobic threshold [*r*^2^ = 0.66, *p* = 0.004; **(C)**].

The OEF within the right striatum at baseline was associated with multiple functional performance, including the test time (*r*^2^ = 0.63, *p* = 0.003, Figure 3A), the relative V_O2max_ (*r*^2^ = 0.51, *p* = 0.007, Figure 3B), and anaerobic threshold (*r*^2^ = 0.66, *p* = 0.004, Figure 3C). Participants with greater OEF within the right striatum at baseline were able to maintain the test for a longer time, and/or had greater anaerobic threshold and relative V_O2max_. Neither the OEFs in other regions nor the CBFs at baseline were associated with those functional outcomes.

**Figure 4:**
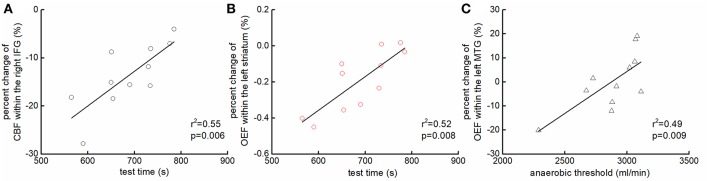
The association between the changes of regional hemodynamics of the brain and functional performance. Participants who had *less* reduction of the CBF within the right inferior frontal gyrus (IFG) [*r*^2^ = 0.55, *p* = 0.006; **(A)**] and/or of the OEF within the left striatum [*r*^2^ = 0.52, *p* = 0.008; **(B)**] were able to maintain the test longer. Additionally, those who had *less* reduction of OEF within the left middle temporal gyrus (MTG) had greater anaerobic threshold [*r*^2^ = 0.49, *p* = 0.009; **(C)**].

The percent change of CBF within the right IFG (*r*^2^ = 0.55, *p* = 0.006, Figure 4A) and the change of OEF within the left striatum (*r*^2^ = 0.52, *p* = 0.008, Figure 4B) was associated with test time. Participants who had *less* reduction of the CBF within the right IFG and/or of the OEF within the left striatum were able to maintain the test longer. Additionally, the anaerobic threshold was associated with the change of OEF within the left MTG (*r*^2^ = 0.49, *p* = 0.009, Figure 4C), such that those with *less* reduction of OEF within the left MTG had greater anaerobic threshold.

## Discussion

The regulation of the blood flow and oxygen extraction in the cerebral regions is the fundamental for the neural activities, which are important for individual's capacity of enduring long-term aerobic exercises. By using advanced fMRI techniques measuring the CBF and OEF of brain regions, our pilot study has demonstrated the first-of-its-kind evidence that compared to the baseline, after the aerobic circle ergometer exercise with the load up to V_O2max_, the cerebral blood flow may decrease globally, and particularly within the left and right striatum, left MTG and right IFG that associate with voluntary motor control, sensory perception, and task motivation; but no significant changes in the global and regional OEFs are observed. Moreover, these neuroimaging metrics, which captures the metabolism of the brain, and their changes after the exercise are associated with the performance of the task (i.e., test time) and the energy consumption (i.e., anaerobic threshold, relative V_O2max_). These preliminary findings may thus provide unique insight into the mechanism underlying the regulation of cerebral hemodynamics pertaining to the aerobic exercise, which are worthwhile to be confirmed in future study of larger sample size.

Studies have shown the benefits of aerobic exercise with mild to moderate physical load on brain health in young and old adults (20, 21). However, the effects of fatiguing aerobic exercise or tasks with high physical load on the functionalities of the brain remain unclear. The cerebral metabolism of oxygen (e.g., the metabolic rate of oxygen) relies on the CBF, OEF and the total oxygen content in the arterial blood (22). Here our results suggest for the first time that the diminished capacity of maintaining the high-load aerobic exercise may be due at least in part to the decreased cerebral blood flow, and the altered ability of these brain regions to extract the oxygen maintains normally.

We observed the significant reduction of CBF within left and right striatum, left MTG and right IFG after the aerobic circle ergometer test. The striatum is the main structure of the basal ganglia, a central hub associated with multiple function, including the control of voluntary movement (23, 24) and task motivation (25, 26). Chaudhuri and Behan (27) have shown that the decreased activation of the basal ganglia alters the neural integrator and the cortical feedback. This dysfunction within the striato–thalamo–cortical loop is associated with the diminished physical function and increased fatigue in many neurodegenerative conditions, such as Parkinson's disease (28). Meanwhile, the MTG is associated with the multisensory integration (29) and the IFG has been linked to the motion inhibition and attention control (30). In our study, a continuous aerobic task with extremely high load up to 100% V_O2max_ was used. The demand of the oxygen supply may thus increase over the maximal supply the vascular system is able to provide. As such, a potential “preserve” mechanism may be initiated: when the exercise is severely overloaded, the supply of oxygenated blood to the basal ganglia region, MTG and IFG decreases, leading to the diminished activation of striatum loop, less transmission of dopamine and declined sensory integration and attention. This helps prevent the body continuing the task of high risk, causing damages to our physiologic systems. Future studies are worthwhile to explore and confirm this potential mechanism by measuring the cerebral changes repeatedly along with the increase of task load.

We also observed that participants with greater resting OEF or less percent reduction in CBF was associated with greater time to maintain the aerobic test, and greater anaerobic threshold and relative V_O2max_. This may indicate that these markers derived from the cerebral hemodynamics are sensitive to the physical and physiologic function. Other studies have demonstrated the effects of exercise on the cerebral function and physical performance (20, 31). Leddy et al. (31), for example, reported that the aerobic exercise restored/enhanced the activation of the cerebral regions (e.g., anterior cingulate gyrus), as well as their physical function in those with post-concussion syndrome. Our study for the first time provide potential links between one's capacity to adapt to the aerobic exercise of high physical load to the cerebral function. The effects of biological aging and other pathological conditions on this relationship are worthwhile to be explored in future's longitudinal studies.

To measure the cerebral oxygen consumption, PET is still the most widely used method. However, it relies on the radiocontrast agent injected into the body (32), and many studies have shown that the radiocontrast agent is toxic and may cause adverse events (33, 34). Meanwhile, the low spatial resolution of other techniques, such as the TCD and NIRs, also limits their applications. We here implemented novel fMRI techniques (i.e., ASL and ASE sequences) to non-invasively measure the CBF and OEF in the brain. These advanced neuroimaging techniques shed light on characterizing the brain in future studies.

The limitation of this pilot study is that the sample size is small (*n* = 11) and currently we focus only on the cohort of elite young athletes. The effects of fatigue on the hemodynamics of the brain in other vulnerary populations, such as those suffering from the chronic fatigue syndrome, are needed to be explored. The observation in this pilot study may still be impacted by the vascular changes within the cerebral regions. Moreover, multiple underlying physiological characteristics may also contribute to the observed changes in CBF here, including the exercise-related changes of adenosine triphosphate, hematocrit, and blood pressure, which, however, was not measured in this pilot study. Additionally, we focused the OEF on only the regions with significant changes in CBF. Future studies of larger sample size are thus warranted to explore and confirm the results of this pilot study by analyzing the regional OEF across multiple brain regions, and to explore the potential physiological pathways through which the fatigue affects the brain's hemodynamics by measuring those metrics. This pilot study nevertheless demonstrated the effects of fatiguing aerobic exercise on the cerebral hemodynamics and the extraction of oxygen in the brain using advanced neuroimaging techniques, revealing a potential preserve mechanism and providing several sensitive neuroimaging markers of physical and physiological function, which may ultimately help the functional assessment in the sports science and clinics.

## Ethics Statement

This study was approved by Institutional Review Board of Beijing Sport University, and conducted according to the principles of the Declaration of Helsinki. All the participants provided written informed consent as approved by the institutional review board.

## Author Contributions

DB, JZ, WJ, YHu, and XW designed the study. DB, YHa, and XY collected the data. JZ and YHa analyzed the data and performed statistical analyses. DB and JZ drafted the manuscript. All authors contributed to and approved the final version.

### Conflict of Interest Statement

The authors declare that the research was conducted in the absence of any commercial or financial relationships that could be construed as a potential conflict of interest.
